# Mdm4: don't judge an isoform by its mRNA levels!

**DOI:** 10.18632/aging.100826

**Published:** 2015-10-20

**Authors:** Boris Bardot, Franck Toledo

**Affiliations:** Genetics of Tumor Suppression (Equipe labellisée Ligue), UMR 3244 IC/CNRS/UPMC, Institut Curie, Paris, France

**Keywords:** Mdm4, p53, cancer, alternative splicing, exon skipping

Alternative splicing of RNA precursors expands the coding capacity of the genome by allowing the production of protein isoforms of distinct and even opposing functions from a single gene. It may also constitute a major mechanism of gene expression regulation. Importantly, pathways frequently deregulated in cancer often play important roles in promoting aberrant splicing, which in turn could affect tumor development by modulating cell cycle control, apoptosis, metabolism, angiogenesis, invasion and metastasis [1].

Inactivation of the p53 pathway is a common step in human malignancy. It often occurs through mutations in the *TP53* gene, or through an overexpression of the *MDM2* or *MDM4* genes, which encode major p53 negative regulators. Interestingly, all three genes were found to produce several isoforms through different mechanisms, including alternative splicing [2, 3]. Some of these isoforms are aberrantly expressed in tumors but their exact role is still largely unknown.

We recently generated a mouse model to address the role of Mdm4-S, a short splice variant of Mdm4. This alternative transcript results from a skipping of exon 6. It encodes a protein that only contains the N-terminal p53-binding domain of Mdm4, followed by a few amino acids of unrelated sequence resulting from a frameshift caused by exon skipping [4]. We focused our attention on this isoform because in humans, increased levels of the MDM4-S transcript were observed in several cancers, and correlated with a bad prognosis.

Previous studies led to opposing views on the significance of this splice variant. Some authors proposed that the MDM4-S transcript encodes a strong p53 inhibitor (stronger than MDM4-FL, the full-length protein), although evidence for an overexpression of the MDM4-S protein in cancer cells remained controversial [4]. On the opposite, other researchers proposed that the main effect of this alternative splicing might be to reduce MDM4-FL expression in tumor cells with mutant p53 or amplified *MDM2* [5]. To unambiguously determine the physiological impact of the Mdm4-S splice variant, we generated a mouse with a targeted deletion of the Mdm4 exon 6, thereby creating an obligatory exon skipping. The mutant allele (*Mdm4^ΔE6^*) prevented the expression of Mdm4-FL, but also led to increased Mdm4-S mRNA levels. We were thus able to determine the consequences of altered Mdm4-S/Mdm4-FL ratios, in animals carrying one or two copies of the *Mdm4^ΔE6^* allele, over various genetic backgrounds of increasing p53 activity (p53^Δ/Δ^, p53^ΔP/ΔP^, p53^+/+^ and p53^+/Δ31^, with the p53^ΔP^ and p53^Δ31^ alleles respectively encoding an hypomorphic mutant protein that lacks the p53 proline-rich domain, or an hypermorphic mutant that lacks the p53 C-terminal domain). All our experiments led us to conclude that the *Mdm4^ΔE6^* allele leads to p53 activation, because the increased expression of the Mdm4-S transcript is not sufficient to ensure a strong expression of the Mdm4-S protein. Hence, the main effect of this splice variant is to reduce Mdm4-FL expression [6].

Almost thirty years after the description of the first p53 splice variant [7], reports of alternative p53, MDM2 or MDM4 transcripts appear to accumulate at an increasing pace. In that respect, our results may serve as a cautionary tale, because they clearly show that an alternative transcript that is overexpressed in cancers, and that may encode a protein isoform that transfection studies had characterized as a potent p53 inhibitor, actually leads to p53 activation in an *in vivo* setting, due to the instability of the encoded protein. Interestingly, this observation may correspond to a very common situation, because a recent estimate suggested that 68% of alternative transcripts that result from exon skipping encode unstable proteins [8].

Importantly, the fact that the Mdm4-S alternative transcript does not encode a stable oncoprotein should not lead to conclude that this splicing event is meaningless for clinicians. At the very least, the overexpressed MDM4-S transcript appears as a useful prognostic marker for tumor progression. Furthermore, for cancer cells in which a WT p53 is often inhibited by an increased expression of Mdm4-FL, strategies to promote exon skipping could be useful to reactivate p53 and inhibit tumor progression (JC Marine, personal communication). Thus, further studies are needed to carefully evaluate the biological impact of the many isoforms of core components of the p53 pathway, because they may suggest new therapeutic strategies against cancer.

## References

[1] David CJ, Manley JL (2010). Genes Dev.

[2] Surget S (2013). Onco. Targets Ther.

[3] Jeyaraj S (2009). Front. Biosci.

[4] Rallapalli R (1999). J. Biol. Chem.

[5] Lenos K (2012). Cancer Res.

[6] Bardot B (2015). Oncogene.

[7] Arai N (1986). Mol. Cell. Biol.

[8] Hao Y (2015). Cell Reports.

